# The role m^6^A RNA methylation is CNS development and glioma pathogenesis

**DOI:** 10.1186/s13041-021-00831-5

**Published:** 2021-07-19

**Authors:** Ting Pan, Fan Wu, Liwen Li, Shiyan Wu, Fang Zhou, Ping Zhang, Caixing Sun, Liang Xia

**Affiliations:** 1 School of the Second Clinical Medical College, Zhejiang Chinese Medical University, Hangzhou, 310053 China; 2 Department of Gynecological Oncology, Cancer Hospital of University of Chinese Academy of Sciences (Zhejiang Cancer Hospital), Institute of Basic Medicine and Cancer (IBMC), Chinese Academy of Sciences, Hangzhou, 310022 People’s Republic of China; 3 Department of Neurosurgery, Cancer Hospital of University of Chinese Academy of Sciences (Zhejiang Cancer Hospital), Institute of Basic Medicine and Cancer (IBMC), Chinese Academy of Sciences, Hangzhou, 310022 People’s Republic of China; 4 Key Laboratory of Head & Neck Cancer, Translational Research of Zhejiang Province, Hangzhou, 310022 People’s Republic of China

**Keywords:** Glioma, GBM, m^6^A methylation, METTL3, WTAP, ALKBH5, FTO

## Abstract

Epigenetic abnormalities play a crucial role in many tumors, including glioma. RNA methylation occurs as an epigenetic modification similar to DNA methylation and histone modification. m^6^A methylation is the most common and most intensively studied RNA methylation, which can be found throughout the RNA life cycle and exert biological functions by affecting RNA metabolism. The m^6^A modification is primarily associated with three types of protease, which are encoded by the writer, eraser and reader genes, respectively. It has been shown that the m^6^A methylation has close connections with the occurrence and development of many tumors, including glioma. In this study, the concept and the research progress of m^6^A methylation are reviewed, especially the role of m^6^A methylation in glioma. Moreover, we will discuss how glioma is paving the way to the development of new therapeutic options based on the inhibition of m^6^A deposition.

## Introduction

Glioma is the most common and most malignant primary intracranial tumor. It is featured by high heterogeneity, extremely poor prognosis, and unclarified molecular mechanism of occurrence and development [1]. It is generally believed that the primary pathogenesis of glioma covers activation of the important proto-oncogenes, inactivation of the tumor suppressor genes, and the dysregulation of the molecular network [2]. The regulation of glioma development at the molecular level has become one of the research priorities.

Epigenetic modification of genes has drawn considerable attention in the studies on tumor pathogenesis. Recently, it has been found that epigenetic abnormalities, including DNA methylation, covalent histone modification, and microRNA abnormalities, play an essential role in the development of tumors [3]. At present, methylation of some genes has been reported to be closely related to the occurrence and progression of glioma and also to the prognosis and chemoradiotherapeutic sensitivity in glioma patients. Promoter methylation of the O^6−^methylguanine DNA methyltransferase (MGMT) gene is mainly investigated in glioma. MGMT is a key DNA repair enzyme, which can reverse the DNA damage caused by the alkylating agents, thereby inducing the resistance to temozolomide (TMZ) and nitrosoureas. The expression level of MGMT is related to the level of promoter methylation [4]. Many studies have indicated that the MGMT expression is downregulated after promotor activation of MGMT, which in turn enhances the response to chemoradiotherapy. A consensus has been reached that the MGMT methylation is closely related to the prognosis, sensitivity to chemoradiotherapy, and treatment decision-making in glioma patients [4].

RNA methylation regulates gene expression at the post-transcriptional level. RNA methylation is considered as an epigenetic modification similar to DNA methylation and histone modification. Several types of RNA (including rRNA, tRNA, snRNA, mRNA and lncRNA) modification have been discovered [5], and N6-methyladenosine (m^6^A) methylation is the most common one. m^6^A methylation is the most common type of RNA modification in eukaryotes. Since its first discovery in the 1970s, m^6^A methylation has been extensively studied in many species, including yeasts, *Arabidopsis thaliana*, fruit flies, mammals, and even in the RNA viruses [6–8]. In recent years, we have witnessed rapid progress in co-immunoprecipitation and RNA sequencing technology, which allow for the identification and localization of the m^6^A methylation sites in mRNAs. These emerging technologies fuel deeper and more extensive researches. So far, m^6^A RNA methylation has been found to play important regulatory roles in tissue growth, circadian rhythm, DNA damage response, and gender determination. More importantly, m^6^A RNA methylation is closely associated with tumor occurrence, development, and treatment. m^6^A methylation has a close relationship with hematologic malignancies and regulates the proliferation and differentiation of normal hematopoietic cells and leukemia cells. This will further affect the sensitivity of leukemia cells to chemotherapeutic drugs, which is significant for the occurrence, development, and drug resistance of acute leukemia [9]. Besides hematologic malignancies, genetic alterations and abnormalities of the m^6^A regulators are also closely related to the malignant progression of many solid tumors, including breast cancer, liver cancer, colorectal cancer, pancreatic cancer, hematologic malignancies, endometrial cancer, cervical cancer and glioma. Some studies have demonstrated that these abnormalities are closely related to the resistance to chemotherapy and radiotherapy [10]. m^6^A methylation has been proved to be reversible, and reversing m^6^A methylation may be a potential approach for anti-tumor treatment. Some small-molecule inhibitors, such as inhibitors of the demethylase FTO, have been applied to treat tumors, implying the potential value of reversing m^6^A methylation in anti-tumor treatment [11]. Here, the concept of m^6^A methylation and its role in the nervous system are reviewed, especially its interaction with the occurrence of glioma, alongside the latest research progress.

## Chemical structure of RNA m^6^A methylation

RNA m^6^A methylation is regulated by the methyltransferase complex, which consists of methyltransferase-like protein 3 (METTL3), methyltransferase-like protein 14 (METTL14), and Wilms tumor 1-associated protein (WTAP). Among them, METTL3 binds to S-adenosyl methionine (SAM) and catalyzes m^6^A methylation. METTL14 primarily serves as the platform for substrate binding. WTAP is responsible for recruiting the METTL3/METTL14 complexes into the nuclei, where they catalyze methylation [12]. The complex, composed of METTL3, METTL14 and WTAP, is also known as a writer. Methylation catalyzed by the writer can be reversed by the eraser (e.g., fat mass and obesity-associated protein) and AlkB homolog 5 (ALKBH5). The methylated mRNAs, which can be recognized by several RNA-binding proteins (e.g., YTHDC1, YTHDC2, YTHDF1, YTHDF2 and HNRNPC) to fulfill the biological functions, are also known as the readers [13, 14]. In the presence of methyltransferase, a nitrogenous base (specifically referring to adenylic acid) undergoes methylation at the nitrogen-6 position, which is known as m^6^A methylation.

## m^6^A methylation in the nervous system

The sequencing results showed that during embryogenesis, the level of RNA m^6^A modification increases significantly [15–18]. As compared with other organs or tissues, the overall level of m^6^A in the brain is considerably higher. This indicates the potential neurobiological functions fulfilled by m^6^A modification in the nervous system, which deserves further investigation [19–23].

**Fig. 1:**
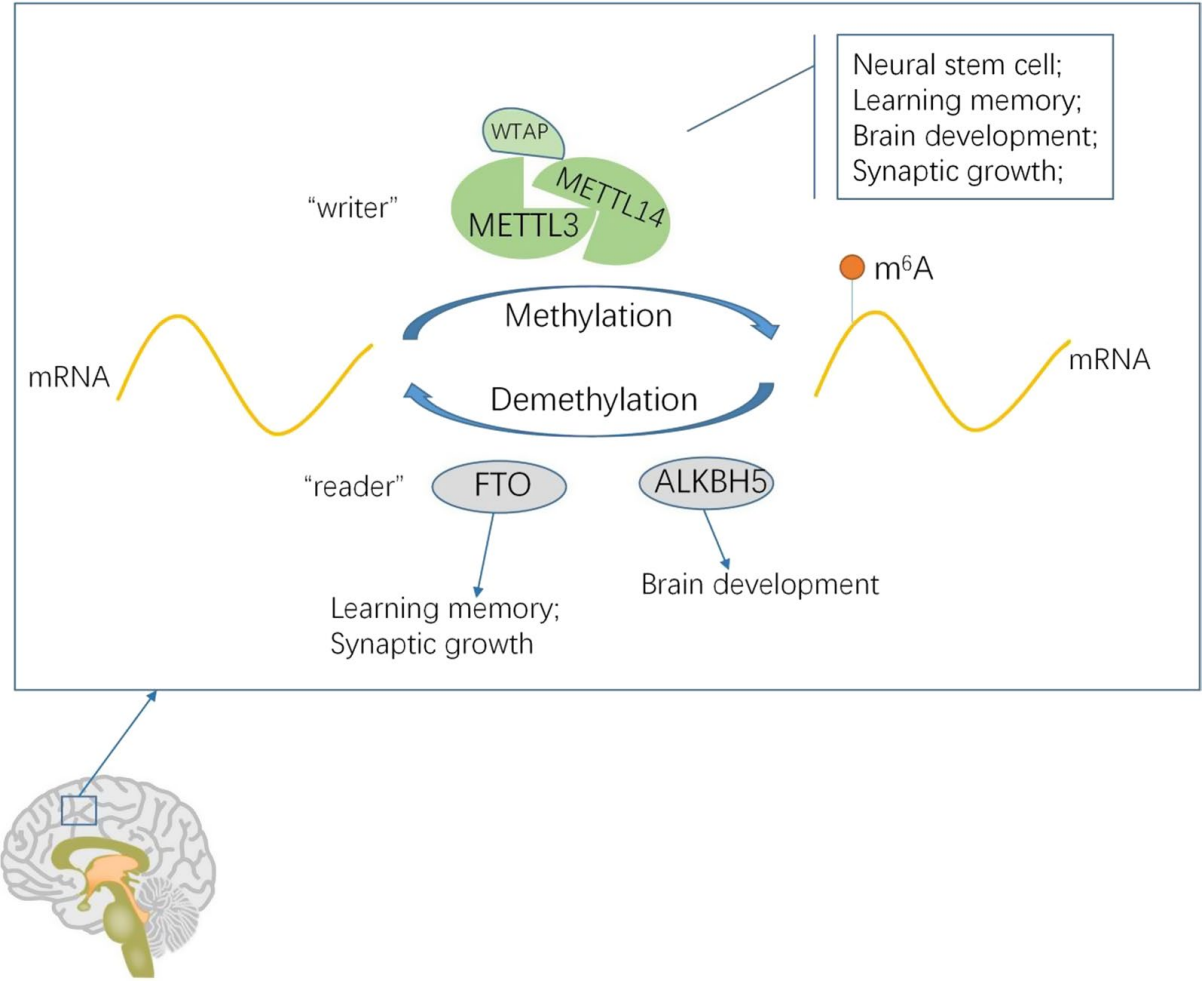
Role of m^6^A in normal brain development [24–37]

A large number of studies have shown that mRNA m^6^A modification may influence the self-renewal and differentiation of the neural stem cells [24–26]. Inactivation of Mettl3 in mouse and human embryonic stem cells leads to a reduction of m^6^A levels, thereby interfering with transition of the neurons from self-renewal to differentiation. Mettl3 knockout can lead to early embryonic death and damage to the formation of mature neurons in the embryoid bodies [18, 27]. Mettl14 and Mettl3 are known to be involved in neurogenesis by regulating the cell cycle progression of the cortical neural stem cells [28]. SMAD2/3 proteins can bind to the METTL3-METTL14-WTAP complexes to promote differentiation of the embryonic stem cells into the endoderm cells [29]. The Ythdf2-mediated mRNA clearance through m^6^A plays a regulatory role in the neurodevelopment of mice. The deficiency of embryonic Ythdf2 severely influences the proliferation and differentiation of neural stem cells [30]. The above evidence demonstrates that m^6^A promotes the proliferation of neural stem cells and prevents premature differentiation of cells, thereby ensuring the reserve of the neural stem cells (Fig. 1).

mRNA m^6^A modification may play a role in learning and memory by regulating the physiological and stress-coping behaviors of the mammalian brains and enhancing fading memories [31–34]. mRNA m^6^A modification plays a crucial part in the death of dopaminergic neurons. It is a newly discovered element in the gene regulatory network specific to the mouse brains [16, 35]. Extensive and dynamic m^6^A methylation has been reported in the developing mouse brains. RNA m^6^A methylation is controlled precisely in both temporal and spatial terms and is involved in the regulation of the brain development of mice after birth [36, 37] (Fig. 1).

## m^6^A roles in glioma

**Fig. 2:**
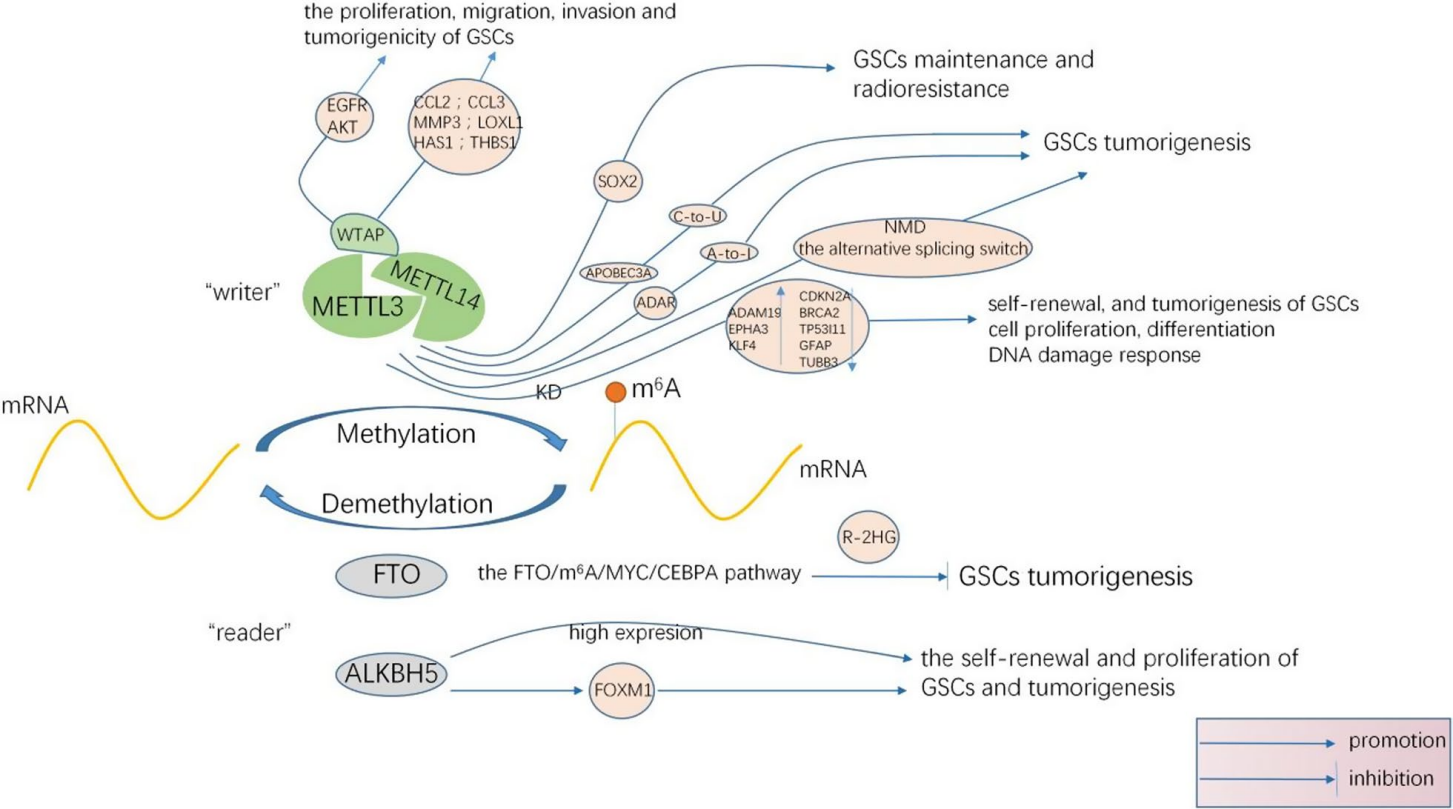
Role of m^6^A in glioma pathogenesis [38–56]

Several studies have revealed the role of m^6^A in glioblastoma (GBM). The m^6^A level in the glioblastoma stem cells (GSCs) considerably affects the growth, self-renewal, and development of tumors. The m^6^A methylation is expected to become the new target for the treatment of GBM (Fig. 2).

### METTL3 and METTL14 in glioma

METTL3, also known as MT-A70, is the first m^6^A methylase ever reported [38]. METTL14 is a heterodimer which belongs to the MT-A70 family and is homologous to METTL3. It has been reported that METTL14 knockdown(KD) in Hela cells decreased the level of m^6^A methylation. This finding demonstrates that METTL14 is an important component of the m^6^A methyltransferase complex [12]. Some studies have shown that METLL3 is a subunit with catalytic activity, while METTL14 is responsible for recognizing substrate. METLL3 and METTL14 form a stable methyltransferase complex at a 1:1 ratio, which catalyzes m^6^A modification of the target RNA [39].

Qi Cui et al. [40] showed that the KD of METTL3 and METTL14, the m^6^A methyltransferases, induced upregulation of the proto-oncogenes (e.g., ADAM19, EPHA3, and KLF4) and downregulation of the tumor suppressor genes (e.g., CDKN2A, BRCA2 and TP53I11), the astrocyte marker GFAP and neuronal marker TUBB3 were also downregulated, thereby promoting self-renewal, and tumorigenesis of GSCs. Then Gene ontology analysis revealed that METTL3 or METTL14 KD regulated the expression of genes involved in important biological processes, including cell proliferation, differentiation, and DNA damage response. In contrast, overexpression of METTL3 caused an upregulation of m^6^A. A high level of m^6^A further led to a reduction in the migration and proliferation capacities of the cells, thus inhibiting tumor growth. In their study, mRNA m^6^A methylation acted as a therapeutic target in GBM. Besides, another study showed that the clinical invasiveness of GBM was correlated with the high expression of METTL3. METTL3 maintained its carcinogenic effect by regulating NMD (Nonsense-mediated mRNA decay), the splicing factor, and the alternative splicing switch in GBM [41]. A study from India found the essential role of METTL3-mediated m^6^A modification in glioma stem-like cells maintenance and radioresistance. RNA immunoprecipitation studies identified SOX2 as a bonafide m^6^A target of METTL3 and the m^6^A modification of SOX2 mRNA by METTL3 enhanced its stability. The recruitment of Human antigen R (HuR) to m^6^A-modified RNA is essential for SOX2 mRNA stabilization by METTL3. METTL3 silenced GSCs showed enhanced sensitivity to γ-irradiation and reduced DNA repair as evidenced from the accumulation of γ-H2AX. Exogenous overexpression of 3′UTR-less SOX2 in METTL3 silenced GSCs showed efficient DNA repair and also resulted in the significant rescue of neurosphere formation from METTL3 silencing induced radiosensitivity. Thus METTL3 was proposed as a potential molecular target for GBM treatment [42]. Some researchers believed that m^6^A methylation in GSCs was primarily mediated by METTL3. The silencing of METTL3 might lead to an abnormal increase in alternative splicing events. It was presumed that the m^6^A reader protein played a crucial role in the functional stability of RNA. METTL3 altered the A-to-I and C-to-U RNA editing events by regulating the RNA-editing enzymes ADAR and APOBEC3A. Similar to the protein-encoding genes, m^6^A-marked lincRNA (long intergenic non-coding RNA) showed METTL3-dependent overexpression. METTL3 played a crucial role in multiple steps of RNA processing and successfully coordinated the execution of the carcinogenic pathway in GSCs [43]. A study based on in silico analyses of the sequencing data obtained in one GSC line silenced for METTL3, they showed that m^6^A methylation was strongly impaired in METTL3 KD cells, thus confirming the primary importance of this enzyme for global m^6^A RNA methylation in GSCs. The KD of METTL3 produced a widespread downregulation of protein coding gene expression, in particular of those genes whose mRNAs contained METTL3-mediated m^6^A sites. In addition, a number of other GSC transcription factors were repressed upon METTL3 deprivation, even if their mRNAs were not affected by m^6^A methylation, indicating, again, that the METTL3 tumoral role in GSCs may go beyond the pool of mRNAs directly affected by its enzymatic activity [43]. The above studies prove that m^6^A methylation of mRNA is closely related to the self-renewal of GSCs and the occurrence of GBM.

### WTAP in glioma

WATP is an indispensable protein for m^6^A methylation [44]. Ping et al. [45] demonstrated that WTAP helped coordinate the localization of METTL3-METTL14 heterodimer to the nuclear speckles, thereby promoting m^6^A methylation. In Sorci et al.’ study [46], either the KDor overexpression of METTL3 led to an overexpression of the WTAP gene. This indicated that the METTL3 gene expression played an important role in the homeostatic regulation of WTAP protein. However, their results showed that in the absence of METTL3, an upregulation of WTAP alone was insufficient to promote cancer cell proliferation. The above data indicate that the carcinogenic effect of WTAP is closely related to the m^6^A methyltransferase complex. Then the next question is, "Is WTAP related to the occurrence and development of cancers?" Xi et al. [47] found that WTAP was overexpressed in GBM. WTAP enhanced the proliferation, migration, invasion and tumorigenicity of GSCs in heterografts by mediating the phosphorylation of epidermal growth factor receptor (EGFR) and AKT. Besides, WTAP is also involved in the regulation of the expressions of some genes related to cancer cell movements, such as chemotactic ligand 2 (CCL2), chemotactic ligand 3 (CCL3), matrix metallopeptidase 3 (MMP3), lysyl oxidase-like protein 1 (LOXL1), hyaluronan synthase 1 (HAS1), and thrombospondin-1 (THBS1) [48]. Overexpression of WTAP is an independent adverse prognostic factor related to age and WHO staging. It usually predicts poor overall survival in GBM patients [47]. Therefore, WTAP may be a prognostic marker in GBM.

### ALKBH5 and FTO in glioma

ALKBH5 is a Fe^2+^- and α-ketoglutarate-dependent non-heme oxygenase and the second discovered demethylase^.^ ALKBH5 is a member of the AIkB family. Unlike other AIkB, ALK⁃BH5 only displayed a demethylation ability on m^6^A in the single-stranded RNA/DNA [49]. ALKBH5, in the presence of the hypoxia-inducible factors (HIFs), increased the stability of NANONGmRNA and upregulated its expression by decreasing the m^6^A methylation in NANONG. Consequently, the conversion from breast cancer cells to tumor stem cells was induced for the use in the research. This finding provides a new pathway to the research on tumor stem cells [50]. Alkbh5 was highly expressed in the whole brain during the embryonic stage, and then declined dramatically during the postnatal and adult stages. In line with the result of Alkbh5, FTO protein levels also showed an age-dependent reduction in the mouse brain [51]. Zhang et al. [52] found that ALKBH5 was overexpressed in the GSCs. Inhibiting ALKBH5 significantly decreased the tumorsphere formation frequency of recurrent GBM-derived GSC11, GSC17 and GSC23 and reduced the expression of Nestin as well as SOX2, Nanog, Oct4, the core transcription factors that endow tumor cells with self-renewal ability, confirming the impact of ALKBH5 on GSC self-renewal. In GSC11, GSC17, and GSC23 cells, loss of ALKBH5 inhibited cell growth and decreased DNA replication. However, depletion of ALKBH5 had no effect on the growth of SW1783 non-CSC glioma cells. KD of ALKBH5 in GSCs increased the proportions of cells in G0/G1 phase whereas decreased the proportions of cells in S and G2/M phase. Collectively, these results suggest that ALKBH5 critically regulates GSC proliferation. In addition, KD of ALKBH5 in GSCs displayed extended survival with a lower rate of tumor formation. In a word, Inhibiting ALKBH5 would decrease the self-renewal and proliferation of GSCs and tumorigenesis. ALKBH5 demethylates FOXM1 nascent transcripts (FOXM1 is a key transcriptional factor in cell cycle regulation and is also crucial for the self-renewal of GSCs and tumorigenesis). ALKBH5 binds to 3'UTR to upregulate FOXM1 expression. FOXM1-AS, as a nuclear lncRNA (antisense nuclear lncRNA), can further promote the interaction between the FOXM1 nascent transcripts and ALKBH5, thereby accelerating this process. FOXM1-AS knockout also disrupts FOXM1 expression and self-renewal of GSC. After depleting ALKBH5 or FOXM1-AS, inducing FOXM1 expression is a salvage against the growth of GSCs, which further proved the critical role of ALKBH5 in the occurrence of GBM [52] (Table 1).

**Table 1:** Role of m^6^A in glioma pathogenesis

Protein	Functional classification	Known role in brain tumor	Molecular mechanism	Refs.
METTL3	m^6^A writer	·The KD of METTL3 or METTL14 enhances GSC's growth, self-renewal and promotes tumor progression. Overexpressing METTL3 Inhibits GSC's growth and self-renewal ·Sustaining oncogenic role ·A potential molecular target ·Indicating RNA processing and coordinating execution of the carcinogenic pathway	·The KD of METTL3 and METTL14, the m^6^A methyltransferases, induced upregulation of the proto-oncogenes (e.g., ADAM19, EPHA3, and KLF4) and downregulation of the tumor suppressor genes (e.g., CDKN2A, BRCA2 and TP53I11),the astrocyte marker GFAP and neuronal marker TUBB3 were also downregulated, promoting self-renewal, and tumorigenesis of GSCs. METTL3 or METTL14 KD regulated the expression of genes involved in cell proliferation, differentiation, and DNA damage response ·Regulating NMD, the splicing factor, and the alternative splicing switch to maintained carcinogenic effect in GBM ·Enhancing the m^6^A methylation of SOX2-3'UTR to improve the stability of SOX2,which promotes the stemness maintenance and radiation resistance of GSCs ·The silencing of METTL3 might lead to an abnormal increase in alternative splicing events	[40] [41] [42] [43]
METTL14	m^6^A writer	Same as METTL3	Same as METTL3	
WTAP	m^6^A writer	·A regulatory subunit of m^6^A ·WTAP expression predicts poor prognosis in malignant glioma patients ·Regulating migration and invasion of glioblatoma cells	·Coordinating the localization of METTL3-METTL14 heterodimer to the nuclear speckles, thereby promoting m^6^A methylation ·Enhancing the proliferation, migration, invasion and tumorigenicity of GSCs in heterografts by mediating the phosphorylation of epidermal growth factor receptor (EGFR) and AKT ·Regulating the expressions of some genes related to cancer cell movements, such as chemotactic ligand 2 (CCL2), chemotactic ligand 3 (CCL3), matrix metallopeptidase 3 (MMP3), lysyl oxidase-like protein 1 (LOXL1), hyaluronan synthase 1 (HAS1), and thrombospondin-1 (THBS1)	[45] [47] [48]
ALKBH5	m^6^A eraser	·ALKBH5 is widely expressed in neurons, decreased during brain development and highly expressed in GSCs	·Inhibiting ALKBH5 would decrease the self-renewal and proliferation of GSCs and tumorigenesis ·ALKBH5 demethylates FOXM1 nascent transcripts. ALKBH5 binds to 3'UTR to upregulate FOXM1 expression. FOXM1-AS, as a nuclear lncRNA (antisense nuclear lncRNA), can further promote the interaction between the FOXM1 nascent transcripts and ALKBH5, thereby accelerating this process. FOXM1-AS knockout also disrupts FOXM1 expression and self-renewal of GSC. After depleting ALKBH5 or FOXM1-AS, inducing FOXM1 expression is a salvage against the growth of GSCs	[51] [52]
FTO	m^6^A eraser	·FTO plays a critical oncogenic role in self-renewal of GSCs and is required for the development of GBM	·R-2HG can inhibit the proliferation/survival of FTO-high tumor cells by targeting the FTO/m^6^A/MYC/ CEBPA pathway	[54] [55]

FTO is a member of the Fe^2+^ and α-ketoglutarate-dependent ALKB family [53]. The FTO gene is localized to chromosome 16q12.2 and is widely expressed at different stages of human development. It is mainly responsible for regulating the speed of fat consumption, promoting overall metabolic rate, and ensuring the energy balance of the body [53]. In 2001, a team led by Prof. He Chuan at the Chicago University confirmed for the first time that in the modification of either DNA or RNA, the FTO protein is a very important demethylase. Its role in the m6A methylation of RNA is non-negligible [11]. This report marked the new era of research on m6A. According to Rui Su [54, 55], FTO as a direct target of R-2HG(R-2-hydroxyglutarate is a co-metabolite resulting from the high level of isocitrate dehydrogenase 1/2 (IDH1/2) mutants) and a main mediator of R-2HG-induced anti-tumor effects. R-2HG binds directly to FTO protein and inhibits its m^6^A demethylase activity, resulting in a significant increase of global m^6^A abundance in R-2HG-sensitive cells, and the effects of R-2HG is FTO-dependent. MYC is a direct and functionally essential target of FTO, and R-2HG treatment or FTO KD increases m^6^A level on MYC mRNA, leading to mRNA decay and MYC down-regulation, and thereby suppression of MYC signaling. FTO transcription is controlled by CEBPA, and CEBPA mRNA is a direct target of FTO and is positively regulated by FTO in an m^6^A-dependent manner, so that there is a positive reciprocal regulation between FTO and CEBPA; as a result, R-2HG treatment can indirectly downregulate expression of both CEBPA and FTO through the FTO/m^6^A/MYC/CEBPA pathway. Marek Bartosovic et al. [56] presented a comprehensive transcriptome-wide analysis of RNA demethylation and uncovered FTO as a potent regulator of nuclear mRNA processing events such as alternative splicing and 3′ end mRNA processing. They showed that FTO binds preferentially to pre-mRNAs in intronic regions, in the proximity of alternatively spliced (AS) exons and poly(A) sites. FTO KD results in substantial changes in pre-mRNA splicing with prevalence of exon skipping events. The alternative splicing effects of FTO KD anti-correlate with METTL3 KD suggesting the involvement of m^6^A. Besides, deletion of intronic region that contains m^6^A-linked DRACH (D = A, G or U; R = A or G; H = A, U or C) motifs partially rescues the FTO KD phenotype in a reporter system. It has revealed for the first time the dynamic connection between FTO RNA binding and demethylation activity that influences several mRNA processing events.

## Conclusions

Malignant progression and high recurrence rate render gliomas the most lethal type of primary brain tumor [57–59]. Epigenetics is one of the research hotspot in recent years. m^6^A is the most intensively studied epigenetic modification, featured by dynamics and reversibility. mRNA m^6^A methylation has a widespread impact on the nervous system. It plays important roles in the self-renewal of neural stem cells, learning and memory, brain development, synaptic growth, and glioma proliferation. Inhibiting the expressions of m^6^A methyltransferase METTL3 or METTL14 can reduce the m^6^A level and promote the tumor formation ability of GSCs in vivo. On the contrary, the overexpression of METTL3 or the use of FTO inhibitor MA2 (a US Food and Drug Administration approved nonsteroidal anti-inflammatory drug) suppresses GSC growth and self-renewal [40]. Both WTAP and ALKBH5 can promote the proliferation and self-renewal of GSCs and tumorigenesis. These important findings open a new era of the targeted treatment of GBM.

Although the role of mRNA m^6^A methylation in cancers has been increasingly understood in recent years, some significant challenges remain unresolved and questions unanswered. A large number of studies have indicated that the m^6^A regulators and the relevant pathways can be used as therapeutic targets. However, there is still a lack of clinical trials with large sample size, and many side effects are not yet clarified. Whether the m^6^A level and its regulators can be used as the potential diagnostic and prognostic markers of tumors depends on their specificity and sensitivity. Further investigations are required in this respect.

Surprisingly, a recent study showed that 13 major regulators of RNA m^6^A methylation were differentially expressed in gliomas with different clinicopathological features. On this basis, a risk signal was derived from the seven regulators of RNA m^6^A methylation. This signal was not only an independent prognostic marker but also a predictor of the clinicopathological features of glioma. This study systematically proved that the regulators of RNA m^6^A methylation were important participants in the malignant progression of gliomas, which may be used in prognostic stratification and the development of therapeutic strategies [60].

At present, RNA m^6^A methylation is believed to play a vital role in the occurrence and development of GBM. In the future, more research is needed to clarify the mechanism of m^6^A methylation to understand its complex regulatory network and to explore its molecular biological mechanism in tumor occurrence and development. RNA m^6^A methylation offers new hope for the precise, targeted therapy of GBM.

## Data Availability

Not applicable.

## Abbreviations

MGMT: Methyltransferase
TMZ: Temozolomide
METTL3: Methyltransferase-like protein 3
WTAP: Wilms tumor 1-associated protein
SAM: S-adenosyl methionine
ALKBH5: AlkB homolog 5
GBM: Glioblastoma
GSCs: Glioblastoma stem cells
NMD: Nonsense-mediated mRNA decay
lincRNA: Long intergenic non-coding RNA
EGFR: Epidermal growth factor receptor
CCL2: Chemotactic ligand 2
CCL3: Chemotactic ligand 3
MMP3: Matrix metallopeptidase 3
LOXL1: Lysyl oxidase-like protein 1
HAS1: Hyaluronan synthase 1
THBS1: Thrombospondin-1
KD: Knockdown
